# CD300f epitopes are specific targets for acute myeloid leukemia with monocytic differentiation

**DOI:** 10.1002/1878-0261.12549

**Published:** 2019-08-20

**Authors:** Edward Abadir, Robin E. Gasiorowski, Kaitao Lai, Fiona Kupresanin, Adelina Romano, Pablo A. Silveira, Tsun‐Ho Lo, Phillip D. Fromm, Marina L. Kennerson, Harry J. Iland, P. Joy Ho, P. Mark Hogarth, Kenneth Bradstock, Derek N.J. Hart, Georgina J. Clark

**Affiliations:** ^1^ Dendritic Cell Research ANZAC Research Institute Sydney Australia; ^2^ Sydney Medical School University of Sydney Australia; ^3^ Department of Haematology Concord Repatriation General Hospital Sydney Australia; ^4^ ANZAC Research Institute Sydney Australia; ^5^ Northcott Neuroscience Laboratory ANZAC Research Institute Sydney Australia; ^6^ Molecular Medicine Laboratory Concord Repatriation General Hospital Sydney Australia; ^7^ Institute of Haematology Royal Prince Alfred Hospital Sydney Australia; ^8^ Immune Therapies Burnet Institute Melbourne Australia; ^9^ Department of Haematology Westmead Hospital Sydney Australia

**Keywords:** acute myeloid leukemia, antibody epitopes, CD300f, cell surface targeting, isoform expression, monoclonal antibodies

## Abstract

Antibody‐based therapy in acute myeloid leukemia (AML) has been marred by significant hematologic toxicity due to targeting of both hematopoietic stem and progenitor cells (HSPCs). Achieving greater success with therapeutic antibodies requires careful characterization of the potential target molecules on AML. One potential target is CD300f, which is an immunoregulatory molecule expressed predominantly on myeloid lineage cells. To confirm the value of CD300f as a leukemic target, we showed that CD300f antibodies bind to AML from 85% of patient samples. While one CD300f monoclonal antibody (mAb) reportedly did not bind healthy hematopoietic stem cells, transcriptomic analysis found that CD300f transcripts are expressed by healthy HSPC. Several CD300f protein isoforms exist as a result of alternative splicing. Importantly for antibody targeting, the extracellular region of CD300f can be present with or without the exon 4‐encoded sequence. This results in CD300f isoforms that are differentially bound by CD300f‐specific antibodies. Furthermore, binding of one mAb, DCR‐2, to CD300f exposes a structural epitope recognized by a second CD300f mAb, UP‐D2. Detailed analysis of publicly available transcriptomic data indicated that CD34^+^ HSPC expressed fewer CD300f transcripts that lacked exon 4 compared to AML with monocytic differentiation. Analysis of a small cohort of AML cells revealed that the UP‐D2 conformational binding site could be induced in cells from AML patients with monocytic differentiation but not those from other AML or HSPC. This provides the opportunity to develop an antibody‐based strategy to target AMLs with monocytic differentiation but not healthy CD34^+^ HSPCs. This would be a major step forward in developing effective anti‐AML therapeutic antibodies with reduced hematologic toxicity.

**Abbreviations**
ADC: antibody–drug conjugate
AML: acute myeloid leukemia
BM: bone marrow
CHO: Chinese hamster ovary
ECL: enhanced chemiluminescence
HSC: hematopoietic stem cell
HSPCs: hematopoietic stem and progenitor cells
mAb: monoclonal antibody
MFI: median fluorescence intensity
PBMCs: peripheral blood mononuclear cells
TCGA: The Cancer Genome Atlas

## 1 Introduction

Intensive chemotherapy with or without allogeneic hematopoietic cell transplant cures a proportion of younger patients diagnosed with AML; however, these therapies are too toxic for the majority of patients (Juliusson *et al*., 2009). Antibody‐based therapies have significantly improved outcomes in other hematologic malignancies, but their development in AML has been limited in comparison (Cuesta‐Mateos *et al*., 2018). Current classification systems of AML depend on recurrent genetic abnormalities to predict prognosis and inform treatment, but the older French‐American‐British (FAB) classification system is still important in predicting surface molecule expression on AML subpopulations (Vardiman *et al*., 2009). The AMLs with monocytic phenotypes are acute myelomonocytic leukemia and acute monocytic/monoblastic leukemia. Acute myelomonocytic leukemia AMLs account for 5–10% of cases across all age groups with a median age of 50 years, while acute monocytic/monoblastic leukemia AMLs occur in any age range but are most common in children (Swerdlow *et al*., 2008). Together, these AML subtypes account for 50% of all AMLs in infants (Masetti *et al*., 2015). A new targeted therapy with reduced hematologic toxicity in AML with monocytic differentiation, including acute myelomonocytic leukemia as well as acute monoblastic and monocytic leukemia, would be a significant development.

The CD33 antibody–drug conjugate (ADC), gemtuzumab ozogamicin, is the only current antibody‐based therapy for AML, after it was reapproved by the FDA in 2017 (Jen *et al*., 2018). More recent ADCs or chimeric antigen receptor (CAR) T cells targeting CD33 have not been successful, primarily due to significant hematologic toxicity in preclinical models and clinical trials (Pizzitola *et al*., 2014; Stein *et al*., 2018). Hematologic toxicity is a difficult challenge to overcome, as many surface molecules found on AML are present on hematopoietic stem and progenitor cells (HSPCs) (Taussig *et al*., 2005). Other well‐described AML targets such as CD123 and CLL‐1 have also been associated with significant hematologic toxicity in preclinical development (Gill *et al*., 2014; Leong *et al*., 2017). Despite extensive genomic and proteomic analyses of AML and HSPC, no ideal surface target for AML has been found (Perna *et al*., 2017).

CD300f is a potential therapeutic target in AML. CD300f is a member of the CD300 immunoregulatory family encoded by a gene complex on human chromosome 17q25 (Clark *et al*., 2009). It is a transmembrane glycoprotein with both inhibitory motifs and PI3K phosphorylation sites in its cytoplasmic region (Alvarez‐Errico *et al*., 2007). Healthy myeloid cells including CD34^+^ HSPC express CD300f, while proteomic and transcriptomic analyses have shown that it is upregulated in AML samples (Korver *et al*., 2009; Strassberger *et al*., 2014). One CD300f monoclonal antibody (mAb) that bound leukemic blasts but not CD34^+^ HSPCs in the majority of AMLs in a small cohort of human samples had some efficacy in a xenogeneic model of AML using the HL‐60 cell line (Korver *et al*., 2009).

Effective therapeutic antibodies targeting well‐defined epitopes mitigate off‐target toxicity. CD300f isoforms are expressed in leukemic cell lines; immunoprecipitation of CD300f from the histiocytic lymphoma cell line U937 identified 53 and 59 kDa proteins (Alvarez‐Errico *et al*., 2004). Four RNA transcripts were initially described (Alvarez‐Errico *et al*., 2004), more are listed in databases, and the current NCBI database lists seven protein isoforms resulting from alternative splicing: NP_620587 (IREM‐1, Isoform 1), NP_001276011 (Isoform 2), NP_001276012 (Isoform 3), NP_001276013 (Isoform 4), NP_001276014 (Isoform 5), NP_001276015 (Isoform 6), and NP_001276016 (Isoform 7). Studies have examined the binding of CD300f mAbs to the canonical CD300f (Isoform 1) expressed by transfected cells, but their binding to other isoforms is undefined. We have confirmed the validity of CD300f as a target on AML in a cohort of 35 AML patients and demonstrated that different CD300f‐specific mAbs recognize independent extracellular epitopes. Further, we showed expression of CD300f isoforms by cell lines and primary AMLs is complex and the different isoform expressed affects mAb binding. This work has defined key differences in the extracellular region of CD300f that will help design novel AML therapeutic antibodies to specific isoforms and minimize hematologic toxicity.

## 2 Materials and methods

### 2.1 Antibodies

CD300f mAbs used were UP‐D1 (mouse IgG1,κ, eFluor 660 conjugate, Jomar), UP‐D2 (mouse IgG1, κ, PE conjugate and purified, BioLegend, San Diego, CA, USA), and 234903 (rat IgG2b, R&D Systems, Minneapolis, MN, USA). An in‐house mAb, DCR‐2 (IgG1,κ), was generated from a mouse immunized with CD300f Chinese hamster ovary (CHO) transfectants and boosted with recombinant human CD300f‐Fc protein (Sino Biologicals, Beijing, China). The polyclonal antibodies used were rabbit antibody to the peptide representing residues 63–92 of the canonical CD300f sequence (CLM‐1, Abcam) and goat anti‐human LMIR3 (leukocyte myeloid inhibitory receptor; gLMIR3, R&D Systems). All antibodies detected epitopes on the CD300f Ig‐like domain. CMRF‐81 (anti‐tetanus toxoid mouse IgG_1_ (Ju *et al*., 2008)) was used as an isotype control.

### 2.2 Cell lines

The myeloid‐derived cell lines HL‐60, U937, HEL, and THP‐1 (all from ATCC) were grown in complete RPMI containing 200 mm glutaMAX, 100U·mL^−1^ penicillin, 100 μg·mL^−1^ streptomycin, and 10% heat‐inactivated fetal bovine serum [all from Thermo Fisher Scientific (Thermo, Melbourne, Victoria, Australia)].

### 2.3 Human samples

Venous blood and bone marrow (BM) samples were obtained, with informed consent, from healthy volunteers collected through the Department of Hematology, Concord Repatriation General Hospital. Cord blood was obtained through the Sydney Cord Blood Bank with ethical approval to use samples that failed banking volume criteria. Mononuclear cells were prepared using density gradient centrifugation over Ficoll‐Paque following the manufacturer's recommendations (GE Healthcare Life Sciences, Uppsala, Sweden). Mononuclear blasts were prepared from excess AML diagnostic samples following patient consent. All consent forms were written and accompanied by participant information sheets. All patient sample protocols conformed to the guidelines set by the Declaration of Helsinki. Table S1 summarizes the characteristics of AML samples. The Concord Repatriation General Hospital Human Ethics Committee approved all protocols. Samples of AML were categorized as having a monocytic differentiation if they met the 2016 WHO criteria of acute myelomonocytic leukemia as well as acute monoblastic and monocytic leukemia by morphology and immunophenotyping, irrespective of genetic abnormalities.

CD14^+^ monocytes were purified from peripheral blood mononuclear cells (PBMCs) labeled with CD14‐FITC (clone M5E2) by cytometric sorting on a BD Influx or with a CD14‐positive selection kit from Miltenyi Biotec. AML samples were phenotyped with the following mAbs from BD Biosciences (Sydney, New South Wales, Australia): CD45‐V500 (clone HI30), CD34‐PE‐CY7 (clone 581), CD38‐V450 (clone HB7), and CD33‐PE (clone WM53). Results were analyzed with flowjo software (Treestar, Ashland, OR, USA). The gating strategy for identifying the blast and leukemic stem cell populations is shown in Fig. S1. SSC^lo^CD45^dim^ blast populations were purified from AML samples labeled with CD45‐V500.

### 2.4 Flow cytometry

Standard protocols were used to stain cells with directly conjugated specific, or isotype control, antibodies as described previously (Clark *et al*., 2016). Unlabeled mouse mAbs were detected with species‐specific Alexa Fluor (AF) 488 or 647F(ab’)_2_ secondary reagents (all from Thermo). Live cells were identified as propidium iodide^‐^ events. Data were collected on either an Accuri C6, Fortessa LSR, or Influx (BD Biosciences).

To determine whether different antibodies bound similar epitopes, we preincubated target cells with saturating amounts of primary antibody in 0.5% BSA/PBS for 30 min on ice. Cells were washed with 0.5%BSA/PBS before incubation with a subsaturating concentration of the test antibody. Experiments were repeated three times. Percent binding of the test antibody was determined from median fluorescence intensity (MFI) by [MFI Test Antibody‐MFI Primary isotype]/[MFI Primary Antibody‐MFI isotype control] x 100. In cross‐blocking experiments using primary samples, CD34^+^ HSPC, lymphocytes, and monocytes all originated from cord blood (CB) PBMC. AML samples were gated with CD45 and CD34 to exclude nonblasts.

### 2.5 Immunoprecipitation and western blots

For immunoprecipitation, 2.5 × 10^7^ cells were biotinylated with Sulfo‐NHS‐Biotin (Thermo) before lysis in M‐PER mammalian protein extraction reagent (Thermo) containing protease inhibitors (Roche, Castle Hill, New South Wales, Australia). Proteins were immunoprecipitated with antibodies bound to Protein G Dynabeads according to the manufacturer's recommendations (Thermo). Samples were resolved through a 4–12% Bis‐Tris Plus gel (Thermo), with or without antioxidant, and transferred to nitrocellulose using an iBlot system (Thermo). Membranes, blocked with 5% BSA/TTBS, were incubated with primary antibody, followed by HRP‐conjugated species‐specific antibody, detected with enhanced chemiluminescence (ECL) reagent (Clarity ECL Kit, Bio‐Rad), and analyzed using a Bio‐Rad ChemiDoc imaging system (Bio‐Rad, Galdesville, New South Wales, Australia). Biotinylated protein was detected with streptavidin–HRP and ECL.

### 2.6 Gene expression analysis

Total RNA was prepared from freshly purified cell populations or cells growing in exponential growth phase using TRIzol reagent as per the manufacturer's instructions (Thermo). Integrity and quantity of extracted RNA were assessed using an RNA 6000 Nano Bioanalyzer (Agilent Technologies, Mulgrave, Victoria, Australia). All RNA used had a RNA integrity number > 8.8. For cDNA, 100 ng of DNase I (Thermo)‐treated RNA was reverse‐transcribed into cDNA using SuperScript III (Thermo). Oligonucleotide primers designed to detect splice variants were checked for specificity by BLAST alignment and are listed in the supplementary material. Gene expression was performed by qPCR on the cDNA using optimized primers and Fast SYBR^®^ Green Master Mix (Thermo). Duplicate samples of cDNA were amplified using a 7500 Fast Real‐time PCR System (ABI). C_T_ values for splice variant amplification were normalized to the *HPRT* endogenous gene and presented as fold changes to a CD14^+^ or U937 cDNA reference sample using the formula: fold change = 2^−ΔΔCT^ (Pfaffl, 2001). Primer efficiencies were all greater than 98%.

### 2.7 Transcriptomic analysis

Healthy bone marrow HSPC (GSE63569 and GSE69239) from seven individuals and The Cancer Genome Atlas (TCGA) acute myeloid leukemia (LAML) data sets from 151 patients were downloaded. Due to differences in treatment, acute promyelocytic leukemia (M3) was removed from the TCGA analysis. Data sets were aligned with STAR RNA‐seq aligner version 2.4 to GRCh38.d1.vd1 genome (Dobin *et al*., 2013). Read quantification was performed with in‐house shell scripts. The exon 3 read positions were chr17: 74704478‐74704517, and the exon 4 read positions were chr17:74703100‐74703141. RKPM was calculated as [(number of target reads)/(total reads/1 000 000)]/(target length in Kb).

### 2.8 Generation of CD300f transfectants

Full‐length CD300f cDNA (Isoform 1) containing an amino‐terminal c‐myc epitope was expressed under the CMV promoter of the pBud vector in CHO cells. Cells expressing high amounts of surface c‐myc were sorted on a BD Influx. Sequences were validated at the Australian Research Genome Facility.

### 2.9 ELISA

The specificity of antibodies for the CD300f Ig‐like domain, and cross‐reactivity with CD300b, was tested by ELISA using recombinant proteins obtained from Sino Biological. Antibodies and appropriate species and isotype controls were incubated with the immobilized recombinant protein, and binding was detected with the relevant HRP‐labeled secondary antibody and OPD.

### 2.10 Primer sequences

The primer and probe sequences were Fw_hHPRT1: 5′AATTATGGACAGGACTGAACGTCTTGCT; Rv_hHPRT1: 5′TCCAGCAGGTCAGCAAAGAATTTATAGC; CD300f^SI4^_F (amplifies exon 4 in Isoform 4 or 6): 5′CACGCCTACCTCCACTACGTTT; CD300f^C^ _F (amplifies exon 4 in Isoforms 1, 2, 3, 5, 7): 5′ATTGACCCAGCACCAGTCACC; CD300f‐Ex4_R (reverse primer to amplify exon 4 in all Isoforms): 5′GGTGGCCGGTCAGAGTTG.

### 2.11 Statistical analysis

Statistical analysis was performed using prism (GraphPad Software, Inc, San Diego, CA, USA). Comparisons between single groups were analyzed with t‐tests. Exon 3 and exon 4 expressions of RNA‐seq data and UP‐D2 binding of AML cell lines and primary samples were analyzed with one‐way ANOVA with multiple comparisons between groups.

## 3 Results

### 3.1 CD300f antibodies bind to primary AML

**Figure 1:**
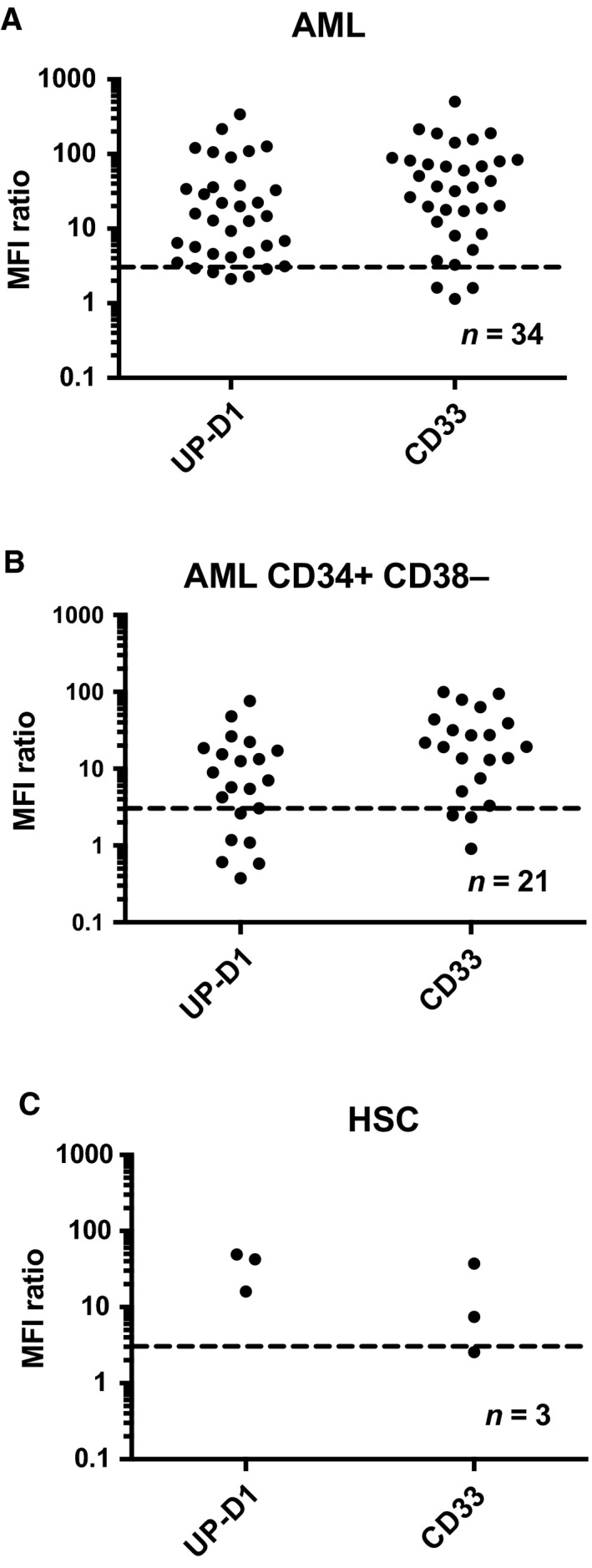
CD300f is expressed on leukemic cells from AML patients. CD300f (UP‐D1) compared to CD33 expression on (A) AML blasts, (B) CD34^+^ CD38^‐^ subset, and (C) Lin^‐^ CD34^+^ CD38^−^ CD45RA ^‐^ CD90^+^ bone marrow HSCs was assessed using multiparameter flow cytometry. The MFI of the population of interest was divided by the MFI of the isotype control to give a MFI ratio. Populations with a MFI ratio ≥ 3, shown above the dotted line, were considered to be positive.

We assessed the binding of the CD300f‐specific mAb, UP‐D1, to 34 newly diagnosed AML samples and healthy bone marrow by flow cytometry using the gating strategy outlined in Fig. S1. UP‐D1 bound to SSC^lo^CD45^dim^ AML blasts in 85% (Fig. 1A) and the SSC^lo^CD45^dim^CD34^+^CD38^−^ in 76% of these patient samples (Fig. 1B). There was no significant difference between the ability of UP‐D1 and anti‐CD33 to bind total AML blasts or the CD34^+^CD38^−^ subset, which is enriched with leukemic stem cells (Fig. 1A,B). UP‐D1 also bound to the Lin^‐^CD34^+^CD38^−^CD45RA^‐^CD90^+^ hematopoietic stem cell (HSC) precursor population within healthy BM (Fig. 1C) similar to CD33. There were no significant differences in the UP‐D1 binding to total CD34^+^ cells, myeloid progenitors, multipotent progenitors (MPPs), or HSC between bone marrow and cord blood (Fig. S1).

### 3.2 Confirmation that CD300f antibodies bind to the CD300f Ig‐like domain

All CD300f protein isoforms listed in NCBI protein database share the CD300f Ig‐like domain but differ in their leader sequence, exon 4‐coded sequence, and their cytoplasmic domain (Fig. S2). We confirmed binding of the CD300f antibodies to CD300f Isoform 1 (Fig. S3A) or Isoform 6 expressed on transfected CHO cells. Because all CD300 molecules share significant amino acid sequence similarity in the Ig domain, we confirmed the specificity of each CD300f mAb to CD300f and not the other family members by either flow cytometry on transfectants or ELISA. Of the CD300 molecules, CD300f shares the highest amino acid sequence identity with CD300b. The CLM‐1 peptide antibody and the gLMIR3 polyclonal antibody bound the Ig domain of CD300b (Fig. S3C).

Each antibody bound to the four CD300f^+^CD300b^‐^ myeloid‐derived cell lines tested, with the exception of the CLM‐1 peptide antibody, which only bound to THP‐1 (Fig. S3D). Each mAb showed a different MFI ratio pattern. UP‐D1 had a low MFI ratio binding to HEL but a high ratio to HL‐60, U937, and THP‐1. The 234903 clone and DCR‐2 mAbs were similar with a high MFI ratio to U937, lower ratio binding to HL‐60 and THP‐1, and an even lower ratio binding to HEL. The mouse UP‐D2 clone and gLMIR3 polyclonal antibody showed similar MFI patterns (Fig. S5). Testing the binding of each antibody to CD300f Isoform 1 expressed on transfectants in the presence of other CD300f demonstrated that no mAb completely cross‐blocked binding of another (Fig. S5). These data indicated the presence of at least four distinct CD300f epitopes recognized by the antibody panel. They are the UP‐D1 epitope, a DCR‐2/243903 epitope, the UP‐D2 epitope, and a CLM‐1 epitope.

**Figure 2:**
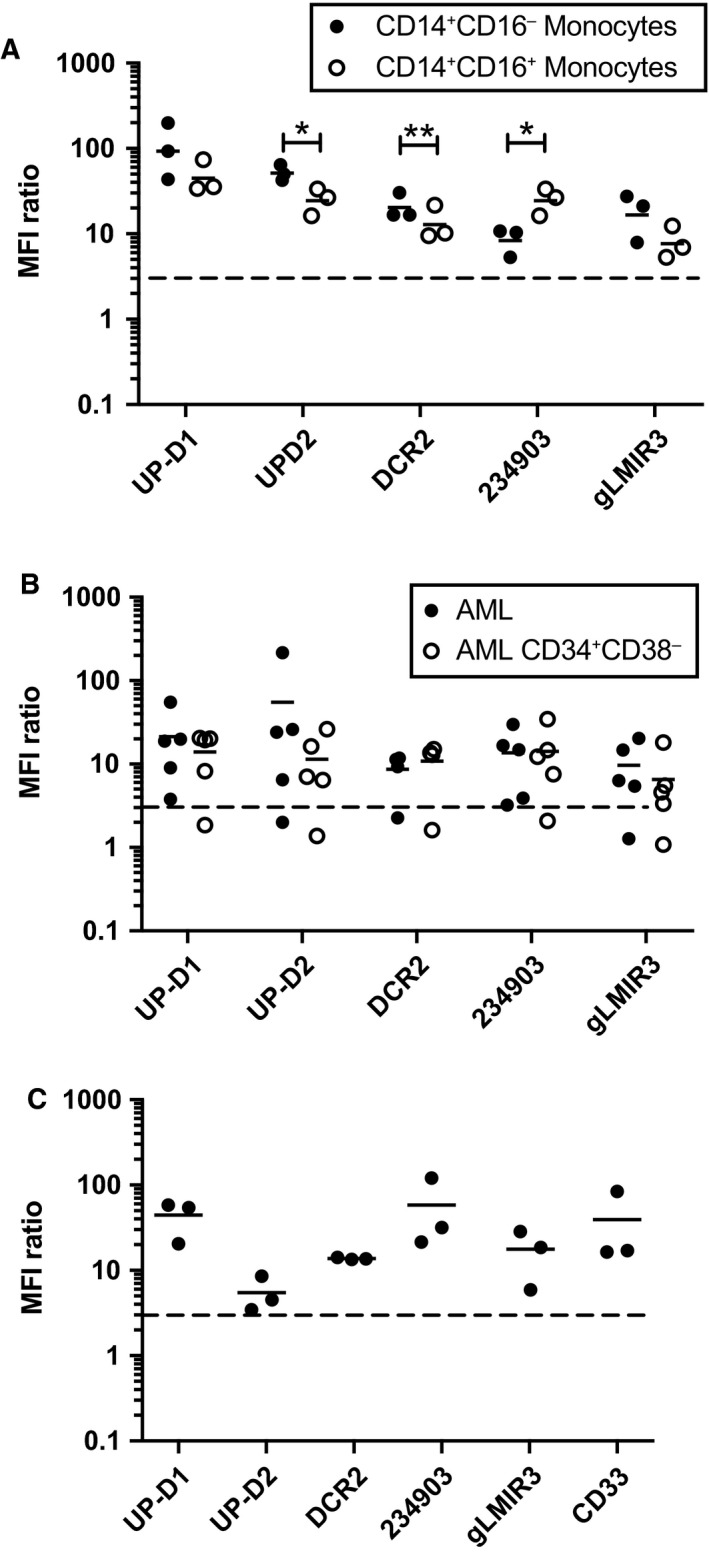
Binding of CD300f antibodies to healthy and leukemic cells. Comparison of CD300f antibodies binding to (A) healthy PBMC populations assessed by multiparameter flow cytometry, (B) AML blasts and CD34^+^ CD38^−^ subset (*n* = 5), and (C) Lin^‐^ CD34^+^ CD38^−^ CD45RA ^‐^ CD90^+^ CB hematopoietic stem cells (*n* = 3). Statistical analysis was performed using *t*‐tests. *P < 0.05 and **P < 0.01.

In healthy PBMC, the four mAbs and gLMIR3 antibody bound to both CD14^+^CD16^−^ (conventional) and CD14^dim^CD16^++^ (inflammatory) monocytes (Fig. 2A). Interestingly, the UP‐D2 and DCR‐2 clones bound to CD14^+^CD16^−^ monocytes with significantly greater intensity than to the CD14^lo^CD16^+^ monocyte population and this pattern was reversed with the 234903 mAb. Having shown that UP‐D1 bound to AML blasts and that it recognized a distinct epitope to other CD300f antibodies, we tested the other antibodies on five representative AML samples (Fig. 2B). All antibodies bound AML blasts, including the CD34^+^CD38^−^ subset (Fig. 2B) except for the CLM‐1 antibody, which bound the blasts from only one sample. The UP‐D1, DCR‐2, and 234903 mAb bound to HSC in healthy CB. There was a weak binding of UP‐D2 (Fig. 2C) and no binding with the CLM‐1 antibody to healthy HSC.

Antigen density of CD300f on the surface of myeloid‐derived cell lines and a number of primary AML samples with a high percentage of blasts was tested using a quantitative bead‐based kit (data not shown). The myeloid‐derived cell lines were expressed in the order of 10^4^ CD300f molecules per cell. Primary AML blasts expressed CD300f at levels ranging from 10^1^ to 10^4^ molecules/cell agreeing with a previous report (Korver *et al*., 2009).

### 3.3 Expression of CD300f isoforms

**Figure 3:**
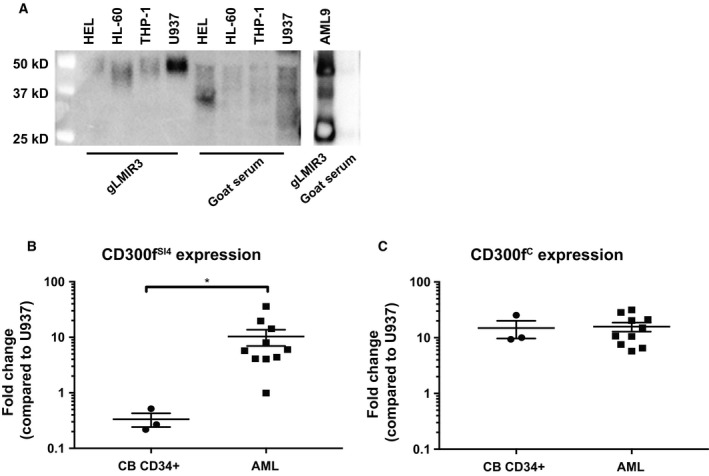
(A) Immunoprecipitation with a CD300f polyclonal antibody reveals multiple forms of CD300f in AML cell lines and primary AML cell samples. CD300f was immunoprecipitated from biotinylated membrane lysates; HEL, HL‐60, U937, THP‐1, and a primary AML9 membrane lysate using goat anti‐LMIR3 or goat serum. Immunoprecipitated protein was detected with streptavidin–HRP and ECL substrate. (B) CD300f exon 4 splice variants or (C) CD300f^C^ was compared between healthy CB‐derived CD34^+^ HSPC and primary AML samples (flow‐sorted or blast count > 90%). Statistical analysis was performed using *t*‐tests. *P < 0.05.

The MW predicted from the CD300f primary sequences ranges from 21.4 kDa to 33.7 kDa. UP‐D1 was originally shown to immunoprecipitate molecules of 53 and 59 kDa (Alvarez‐Errico *et al*., 2004). We used the gLMIR3 antibody to immunoprecipitate CD300f from the myeloid‐derived cell lines and identified proteins with MW ranging from 40 kDa on HL‐60 to 55 kDa on U937 with one predominant band in each cell line (Fig. 3A). Immunoprecipitated CD300f proteins from primary AML lysates that expressed high levels of CD300f had MW of 28 kDa, 40 kDa, and 60 kDa.

Both alternative splicing events and different post‐translational modifications are likely to contribute to the variation in isoform expression. To understand the effect the expression of different isoforms has on antibody targeting of CD300f^+^ cells, we looked in detail at the different extracellular portion of the isoforms. In the databases, the extracellular sequences for CD300f have two forms. The canonical form of CD300f does not express exon 4, and here, it will be identified as CD300f^C^. CD300f Ig domain, encoded by exon 3, spliced to the transmembrane region is present in protein Isoforms 1, 2, 3, 5, and 7 (Fig. S2). The alternative has a splice insertion of exon 4 after the Ig domain before the transmembrane domain. Exon 4 encodes a Ser‐Thr‐rich sequence and is found in protein Isoforms 4 and 6 and here will be referred to CD300f^SI4^.

### 3.4 Differential expression in CD300f Exon 4 between Healthy CD34^+^ HSPC and AML

The qPCR was used to amplify CD300f^SI4^ and CD300f^C^ transcripts from cDNA prepared from myeloid‐derived cell lines, healthy monocytes, healthy CB CD34^+^ HSPC, and AML blasts (Fig. S4). We observed that CD300f^C^ transcripts were more abundant than CD300f ^SI4^ present in healthy CB CD34^+^ HSPC. AML blasts expressed both CD300f ^SI4^ and exon CD300f^C^ transcripts (Fig. 3). There was a significant difference in CD300f ^SI4^ expression between the CB CD34^+^ and AML cells (which were primarily of subtypes with monocytic differentiation) (*P* < 0.05).

**Figure 4:**
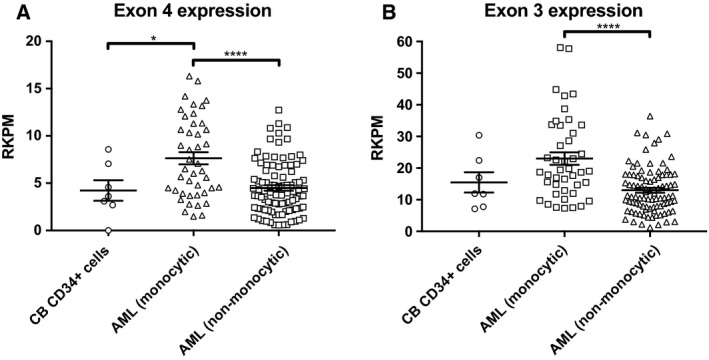
RNA‐seq of CD300f exon 4 between BM CD34^+^ HSPC, monocytic AML, and nonmonocytic AML. Publicly available healthy BM CD34^+^ HSPC (*n* = 7) and AML (*n* = 131) data were analyzed for CD300f exon 4 and exon 3 expressions. (A) CD300f exon 4 expression comparison between BM CD34^+^ HSPC, monocytic AML, and nonmonocytic AML. (B) CD300f exon 3 expression comparison between BM CD34^+^ HSPC, monocytic AML, and nonmonocytic AML. Statistical analysis was performed with one‐way ANOVA with multiple comparisons between groups. *P < 0.05 and ****P < 0.0001.

To confirm our qPCR findings on a larger sample set, we compared publicly available RNA‐seq data from healthy bone marrow CD34^+^ HSPC with AML RNA‐seq data from TCGA project. Exon 4 and exon 3 (expressed on all isoforms) sequences were compared across the bone marrow CD34^+^ HSPC and both monocytic AML and nonmonocytic AML (Fig. 4). There were significant differences in exon 4 but not exon 3 expression between bone marrow CD34^+^ HSPC and monocytic AML (*P* = 0.033). There were significant differences between monocytic and nonmonocytic AML phenotypes in both exon 4 (*P* < 0.001) and exon 3 (*P* < 0.001) expressions.

### 3.5 CD300f antibodies showing different binding to CD300f isoforms

**Figure 5:**
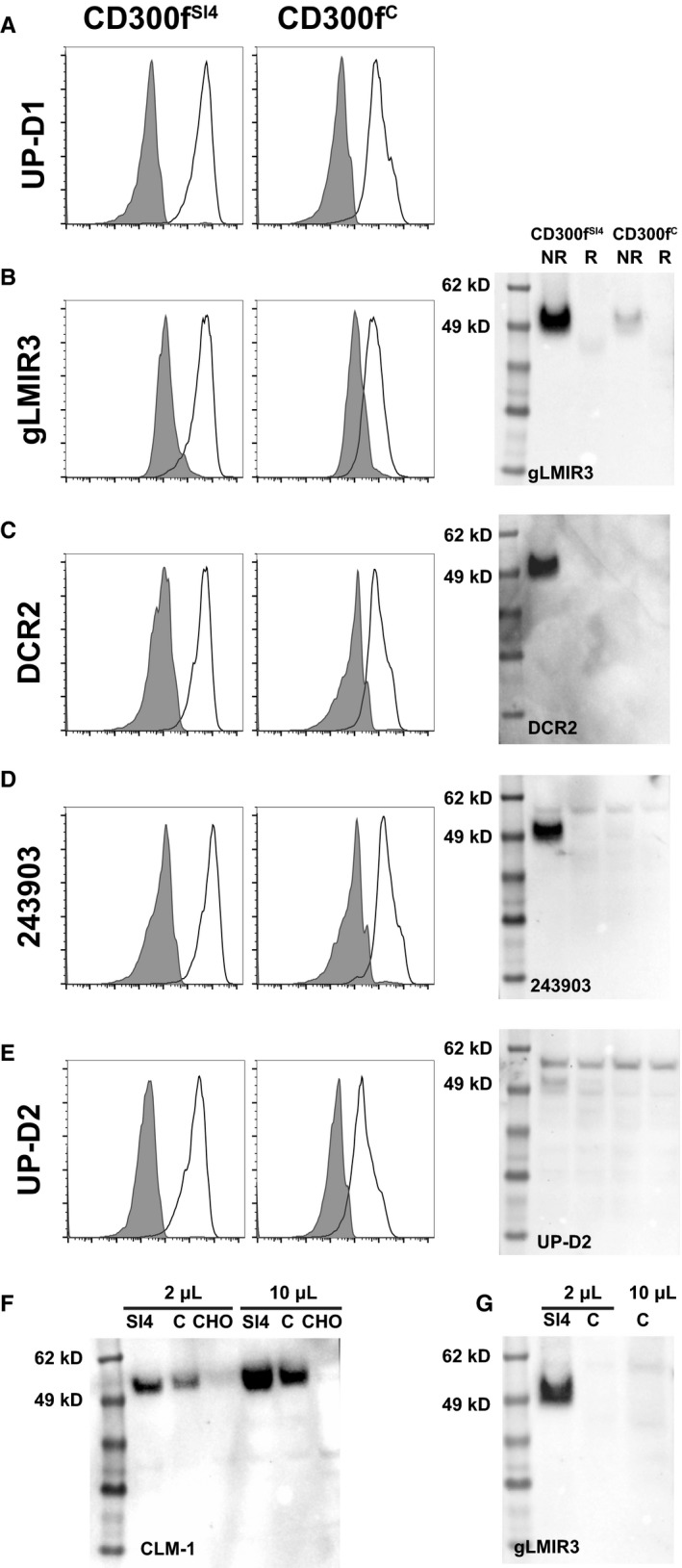
Influence of CD300f exon 4 on antibody binding. The binding properties of anti‐CD300f antibodies (A–E) by flow cytometry and western blot were compared using CHO cells transfected with either the CD300f^SI^ ^4^ present or CD300f^C^. (F) Western blot using CLM‐1 demonstrating the presence of CD300f in both CHO transfectants. (G) Western blot using gLMIR3 at different concentrations to confirm the absence of binding to reduced CD300f^C^‐transfected CHO cells.

All CD300f Abs bound to the CD300f^SI4^ and CD300f^C^ extracellular region when expressed in CHO cells. Comparing the MFI ratios of UP‐D1 to each isoform revealed that UP‐D1 bound to the CD300f ^SI4^ isoform with a threefold higher ratio compared to the CD300f^C^. On the other hand, there was less than twofold difference in UP‐D2, 234903, and DCR‐2 binding to CD300f ^SI4^ isoform compared to CD300f^C^ isoform (Fig. 5). Western blot analysis (Fig. 5) revealed that gLMIR3, DCR‐2, and 234903 Abs bound to a 49kD protein from the CD300f ^SI4^ in nonreducing conditions, but there was little binding in reducing conditions, whereas UP‐D2 worked poorly on western blot under either condition. There was minimal binding to the CD300f^C^ isoform in either reducing or nonreducing conditions by gLMIR3 (Fig. 5G), even when 10‐fold levels of lysate were analyzed. CLM‐1 antibody confirmed CD300f^C^ expression in all samples (Fig. 5F). The presence of the exon 4‐coded Ser‐Thr‐rich sequence alters the structure of the CD300f increasing the exposure of the epitope for each mAb.

### 3.6 DCR‐2 mAb binding reveals the UP‐D2 epitope

**Figure 6:**
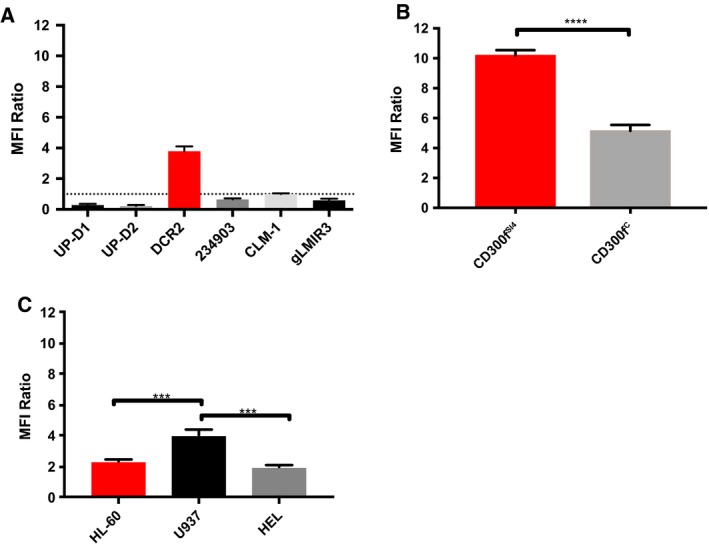
Cross‐blocking studies with DCR‐2 and UP‐D2. Cells were incubated at the saturation point of a primary antibody or an equal concentration of an isotype control and then stained with UP‐D2 PE at 80 ng·mL^−1^. The MFI ratio is the comparison between primary antibody and isotype control groups. (A) The change in UP‐D2 PE binding to CD300f^C^ CHO transfectants with different primary antibody staining. (B) Difference in UP‐D2 binding to CD300f^SI^ ^4^‐ and CD300f^C^‐transfected CHO cells, which were incubated with a saturating amount of DCR‐2 or isotype control prior to UP‐D2 PE. (C) Difference in UP‐D2 binding to AML cell lines, which were incubated with a saturating amount of DCR‐2 or isotype control prior to UP‐D2 PE. Error bars represent SEM. Panel B was analyzed using a t‐test. Panel C was analyzed using a one‐way ANOVA with multiple comparisons between groups. ***P < 0.001 and ****P < 0.0001.

**Figure 7:**
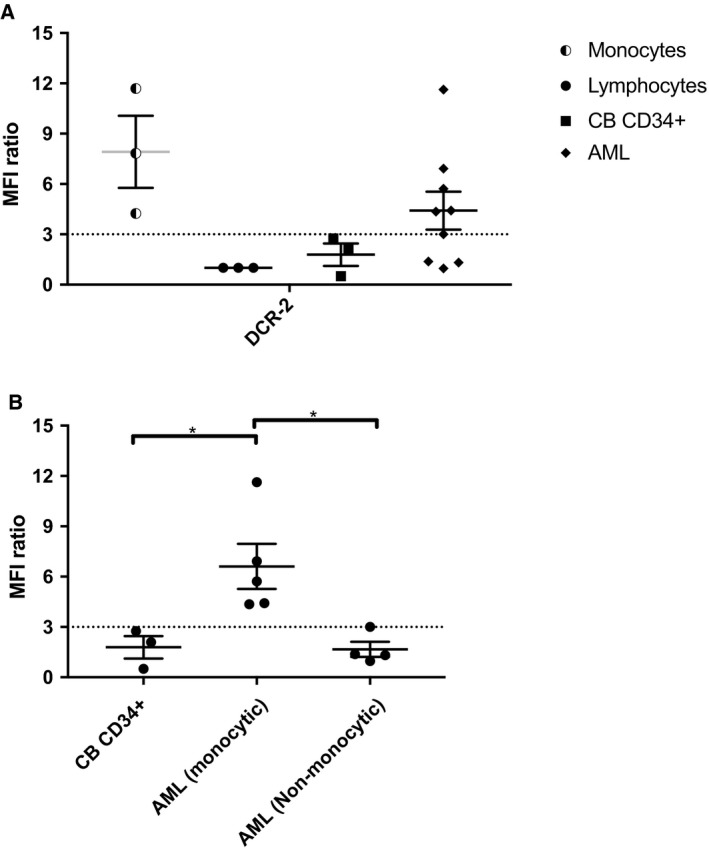
Binding of DCR‐2 to CD300f^+^ cells enhances the binding of UP‐D2 to monocytes, monocytic AML, but not CD34^+^ HSPC or nonmonocytic AML. CB or primary frozen AML cells were incubated with PBS, the saturation point of DCR‐2 (10 μg·mL^−1^), or an equal concentration of an isotype control. Following primary incubation, samples were stained with UP‐D2 PE at 80 ng·mL^−1^. Data for monocytes, lymphocytes, and CD34^+^ HSPC were obtained from CB. (A) Difference in UP‐D2 PE binding across cell types when saturated with DCR‐2 or isotype control, compared to PBS. (B) Differences in UP‐D2 PE binding across CD34^+^ cells between CB, monocytic AML, and nonmonocytic AML. Error bars represent SEM. Statistical analysis was performed with one‐way ANOVA with multiple comparisons between groups. *P < 0.05.

Cross‐blocking experiments demonstrated that no CD300f mAbs completely blocked the binding of another CD300f mAb to CD300f (Figs 6 and S3). Interestingly, in these experiments we observed that instead of blocking, DCR‐2 significantly enhanced the binding of UP‐D2 to CD300f^C^ expressed on CHO cells (Fig. 6). The enhanced binding of UP‐D2 by DCR‐2 was further augmented on CD300f ^SI4^ expressed on CHO cells. This enhancement of UP‐D2 binding was also observed on several AML cell lines. Notably, compared to HL‐60 and HEL cells, U937 cells which express more CD300f ^SI4^ by qPCR also showed the most enhanced binding (Fig. S4). Finally, in primary cells, DCR‐2 significantly enhanced UP‐D2 binding on monocytes but not CD34^+^ HSPC from CB. There was no significant difference in UP‐D2 binding with primary staining using PBS or an isotype control (data not shown). This demonstrated that the isoform of CD300f expressed by HSPCs can be distinguished from that expressed on mature monocytes (Fig. 7). The effect of DCR‐2 on the binding of UP‐D2 was tested on AML samples. Enhancement was evident on AMLs with monocytic differentiation (*P* = 0.0314) but absent on other AMLs compared to CB CD34^+^ HSPC (Fig. 7).

## 4 Discussion

The development of new antibody‐based therapeutics requires the identification of appropriate cell surface protein targets. Our work demonstrates the possibility to develop antibodies to CD300f as therapeutics against AMLs with monocytic differentiation also have the potential for reduced hematologic toxicity compared to currently studied targets. We identified CD300f as a member of the CD300 gene family (Alvarez‐Errico *et al*., 2004; Clark *et al*., 2009), and data from Korver *et al*. suggested CD300f was a potential AML target for both unconjugated antibodies and ADC (Korver *et al*., 2009). Our early analysis (Modra *et al*., 2006) and Korver's data obtained with their CD300f‐specific mAb (clone D12) confirmed expression on the surface of AML cells. This present work has further established the validity of CD300f as a target on AML. A high percentage of AML patients express cell surface CD300f on their blast cells, and the percentage of CD300f^+^ AMLs or CD33^+^ AMLs is similar. Our studies distinguished the expression of CD300f extracellular isoforms that included alternatively spliced variants of the extracellular exon 4‐encoded sequence.

The great need for new AML therapeutics has brought several potential antibody targets into consideration. In addition to CD33, potential targets identified by proteomic and transcriptomic studies of myeloid cell lines and AML samples include CD123, CD96, CD44, CD47, CD32, CLL‐1, IL1RAP, TIM‐3, and LILRB4 (Dobrowolska *et al*., 2013; Gasiorowski *et al*., 2014; Mardis, 2014; Strassberger *et al*., 2014). All these molecules are expressed to some degree by normal cells of the myeloid lineage and bone marrow HSPC raising the possibility of therapeutic antibody hematologic toxicity. To date, it has been difficult to determine an AML‐specific epitope and careful evaluation of prospective therapeutic mAbs for their therapeutic index of activity against AML versus normal hematopoietic precursors is essential. Potential therapeutic mAb to CD300f will need careful evaluation of their specificity with testing for wider CD300 molecule ‘off‐target’ effects. The success of gemtuzumab ozogamicin suggests there is a ‘therapeutic window’ whereby ADC targeting myeloid antigens can exert an antileukemic effect without excessive myelosuppression. The excellent efficacy of gemtuzumab ozogamicin in acute promyelocytic leukemia (Breccia and Lo‐Coco, 2011) which is CD33‐dense suggests that the therapeutic window is likely to be widest when AML subtypes are chosen with high receptor levels.

AML target molecules will inevitably have wider myeloid cell expression. CD300f is expressed primarily within the myeloid cell series and is present on the myeloid cell populations of healthy PBMC (Alvarez‐Errico *et al*., 2004). Three CD300f mAbs bound CD34^+^CD38^−^ BM‐derived HSPC in healthy BM or CB and, importantly, to AML. Korver *et al*. did not detect CD300f on CD34^+^CD38^−^ BM‐derived HSPC using their IREM‐1 mAbs (Korver *et al*., 2009). This difference emphasizes the importance of our observations, which have defined the role multiple isoforms of CD300f will play as potential targets and the fact that different CD300f antibodies clearly target at least four different epitopes.

Our work suggests two possible ways to exploit targeting CD300f in future antibody‐based therapies against AML with monocytic differentiation. The first method would be to generate a mAb, ADC, or other antibody‐based therapeutic derivative that binds preferentially to an exon 4‐related epitope. A second way would be to develop chimeric or humanized versions of DCR‐2 and UP‐D2 for combination therapy in which UP‐D2 could be conjugated with a toxic payload as an ADC or developed into another form of therapeutics. These strategies may result in prolonged monocytopenia. The immunological consequences of a prolonged monocytopenia are unclear, those with germline GATA2 mutations associated with monocytopenia have a higher incidence of opportunistic infections, but these mutations cause additional NK‐ and B‐cell cytopenias (Hsu *et al*., 2011). The significant enhancement of binding to AML with monocytic differentiation would likely lead to a wider therapeutic window than currently seen. A widened therapeutic window would reduce hematologic toxicity by limiting depletion of HSPC. Either method requires further development to test with an expanded cohort of healthy HSPC and AMLs.

Detailed epitope mapping of the rituximab target found that most CD20 Abs bind one of two overlapping epitopes (Klein *et al*., 2013). Our studies identified five immunogenic CD300f epitopes. Crystallization studies of the CD300f Ig domain showed an Ig V‐like domain with a CDR3 region that was structurally variable, and a protrusion from the Ig structure created by a second disulfide bond between Cys54 and Cys62 (Marquez *et al*., 2007). This may explain differences in the ability of polyclonal antibodies to bind more readily to nonreduced compared to reduced forms of CD300f.

The descriptions of CD300f as IREM‐1 identified the canonical CD300f and three splice variants, all of which were missing exon 4 (Alvarez‐Errico *et al*., 2004). Our molecular analysis identified CD300f^SI4^ and CD300f^C^ transcript variants that were differentially expressed in both AML and HSPC. The more marked differential in expression of CD300f^SI4^ on AML compared to HSPC using qPCR as compared to the RNA‐seq analysis may be due to both techniques and samples that were used. A final functional examination of the difference in CD300f^SI4^ would require a mAb that preferentially binds the exon 4 region of CD300f.

While exon 4 does not contribute to the mAb‐binding Ig domain, it contributes to the tertiary structure of the molecule on the cell surface. The fourteen residues coded by the inserted sequence include 2 serine and 7 threonine residues, which have potential to undergo significant post‐translational modification by either O‐linked glycosylation, phosphorylation, or acetylation. Our studies validated the binding of CD300f antibodies to CD300f ^SI4^ and CD300f^C^ transfectants but highlighted that each antibody bound differently to the two forms of CD300f.

CD300f was identified as a potential AML target by independent proteomic, transcriptomic, and empirical investigation of its myeloid‐restricted expression. Our data describing multiple epitopes on the canonical CD300f isoform advance the process of antibody development to CD300f. Understanding how CD300f variant expression relates to changes in splicing mechanisms common in AML (Adamia *et al*., 2014) is critical, and future genetic analysis may predict for CD300f expression.

## 5 Conclusions

The novel finding that CD300f variants resulting from splicing events are more abundant in AMLs with monocytic phenotypes compared to HSPC opens opportunities for a wider treatment window compared to currently tested surface molecules in this subset of AML. The potent effect of DCR‐2 binding revealing a conformational epitope has novel targeting prospects for AML with monocytic phenotypes that should be further explored.

## Acknowledgements

We would like to acknowledge Elizabeth Newman and Christine Fong from the Department of Haematology Concord Repatriation General Hospital for the collection of venous and bone marrow samples and the Sydney Cord Blood Bank for provision of CB. We would like to acknowledge Donna Bonnici for administrative assistance. This work was supported by the Cancer Institute New South Wales Translational Program Grants (11/TPG/3‐02 and 2017/TPG002) and the Australian Government National Health Medical Research Council Program Grant (543727). R.E.G received an NHMRC Scholarship APP1055892, and E.A. is a recipient of an NHMRC Scholarship (APP1134202).

## Conflict of interest

GJC is a Director of DendroCyte BioTech Pty Ltd. GJC and REG are listed as inventors on patents protecting DCR‐2. Other authors have no conflict of interest to disclose.

## Author contributions

EA, REG, FK, PAS, T‐HL, PDF, and GJC performed experiments; EA, REG, HJI, PJH, and KB provided samples and intellectual input; EA, REG, PAS, and GJC analyzed results and made figures; PMH and DNJH contributed intellectually to the project design; EA, REG, and GJC contributed to project design, analysis of results, and writing of manuscript.

## Supporting information


**Table S1.** Clinical characteristics of AML samples tested. ND Not determined.

**Fig. S1.** Gating strategy to identify AML and HSCs. (A) After initially gating on PI negative viable cells, hematopoietic stem cells were identified as lineage‐CD45^dim^CD34^+^CD38^−^CD45RA^‐^CD90^+^. Multipotent progenitors (MPP) were identified as lineage‐CD45^dim^CD34^+^CD38^−^CD45RA^‐^CD90^−^. Myeloid progenitors are contained in the CD34^+^ CD38^+^ subset. (B) Blasts were identified as CD45^dim^SSC^low^. The leukemia stem cell enriched CD34^+^CD38^−^ fraction was identified from this gate. (C) The relative MFI ratios of total CD34 + cells, myeloid progenitors (CD34^+^ CD38^+^ subset), MPP and HSC were compared between bone marrow and cord blood cells.

**Fig. S2.** Sequences of the CD300f isoforms listed in NCBI indicating the alternating exon structure in blue/black type.

**Fig. S3.** Specificity of CD300f antibodies. (A) Binding of CD300f antibodies to CD300f transfected CHO cells. Antibody (unshaded histogram) compared to isotype for each antibody (shaded histogram). CD300f antibodies were tested by ELISA for binding to (B) CD300f‐Ig fusion protein and (C) CD300b‐Ig fusion protein. ELISA was performed *n* = 2, error bars represent SEM from duplicate wells of representative result. (D) Graphs showing the geometric MFI mean of CD300f antibodies binding to four myeloid derived cell lines. Error bars represent SEM.

**Fig. S4.** Expression of multiple CD300f splice variants in primary AML samples. Graph showing fold difference in expression of CD300f^C^ and CD300f^SI4^ in cDNA prepared from AML samples with a high blast count, blasts sorted from AML populations, healthy CD14 monocytes HL‐60, HEL and U937. CD300f specific amplicons were normalized to the HPRT endogenous gene transcripts in U937. Error bars represent SEM.

**Fig. S5.** Cross‐blocking studies with CD300f antibodies. CD300f transfected CHO cells were incubated with a saturating amount of each primary antibody, (x axis). Cells were then stained with the test antibodies (A) UP‐D1 (B) 234903 (C) CLM‐1 (D) LMIR3. The binding of the test antibody in the presence of the primary antibody was calculated as by MFI compared to an isotype control, with 0 binding indicating complete overlap of epitopes, and 1 binding indicating no overlap of epitopes (*n* = 3). Error bars represent SEM.

## Data Availability

The following publicly available data sets were used for this project: GSE63569, GSE69239, and the TCGA LAML.
