# Supplementary material for: CD300f epitopes are specific targets for acute myeloid leukemia with monocytic differentiation
Source: Mol Oncol. 2019 Aug 20;13(10):2107–20. doi: 10.1002/1878-0261.12549 (PMC6763785; doi:10.1002/1878-0261.12549)
Supplement: Supplementary file 2 — Fig. S1. Gating strategy to identify AML and HSCs. (A) After initially gating on PI negative viable cells, hematopoietic stem cells were identified as lineage‐CD45dimCD34+CD38−CD45RA‐CD90+. Multipotent progenitors (MPP) were identified as lineage‐CD45dimCD34+CD38−CD45RA‐CD90−. Myeloid progenitors are contained in the CD34+ CD38+ subset. (B) Blasts were identified as CD45dimSSClow. The leukemia stem cell enriched CD34+CD38− fraction was identified from this gate. (C) The relative MFI ratios of total CD34 + cells, myeloid progenitors (CD34+ CD38+ subset), MPP and HSC were compared between bone marrow and cord blood cells. [file MOL2-13-2107-s002.docx]

Canonical 1 MPLLTLYLLLFWLS---GYSIVTQITGPTTVNGLERGSLTVQCVYRSGWETYLKWWCRGAIWRDCKILVKTSGSEQEVKR 77

Isoform 2 1 MWLPQLDLMRVISAKSQ**G**YSIVTQITGPTTVNGLERGSLTVQCVYRSGWETYLKWWCRGAIWRDCKILVKTSGSEQEVKR 80

Isoform 3 1 MPLLTLYLLLFWLS---**G**YSIVTQITGPTTVNGLERGSLTVQCVYRSGWETYLKWWCRGAIWRDCKILVKTSGSEQEVKR 77

Isoform 4 1 MPLLTLYLLLFWLS---**G**YSIVTQITGPTTVNGLERGSLTVQCVYRSGWETYLKWWCRGAIWRDCKILVKTSGSEQEVKR 77

Isoform 5 1 MWLPQLDLMRVISAKSQ**G**YSIVTQITGPTTVNGLERGSLTVQCVYRSGWETYLKWWCRGAIWRDCKILVKTSGSEQEVKR 80

Isoform 6 1 MWLPQLDLMRVISAKSQ**G**YSIVTQITGPTTVNGLERGSLTVQCVYRSGWETYLKWWCRGAIWRDCKILVKTSGSEQEVKR 80

Isoform 7 1 MPLLTLYLLLFWLS---GYSIVTQITGPTTVNGLERGSLTVQCVYRSGWETYLKWWCRGAIWRDCKILVKTSGSEQEVKR 77

Isoform 1 78 DRVSIKDNQKNRTFTVTMEDLMKTDADTYWCGIEKTGNDLGVTVQVTIDP**A**--------------PVTQEETSSSPTLTG 143

Isoform 2 81 DRVSIKDNQKNRTFTVTMEDLMKTDADTYWCGIEKTGNDLGVTVQVTIDP**A**--------------PVTQEETSSSPTLTG 146

Isoform 3 78 DRVSIKDNQKNRTFTVTMEDLMKTDADTYWCGIEKTGNDLGVTVQVTIDP**A**--------------PVTQEETSSSPTLTG 143

Isoform 4 78 DRVSIKDNQKNRTFTVTMEDLMKTDADTYWCGIEKTGNDLGVTVQVTIDP**A**stpapttptsttftPVTQEETSSSPTLTG 157

Isoform 5 81 DRVSIKDNQKNRTFTVTMEDLMKTDADTYWCGIEKTGNDLGVTVQVTIDP**A**--------------PVTQEETSSSPTLTG 146

Isoform 6 81 DRVSIKDNQKNRTFTVTMEDLMKTDADTYWCGIEKTGNDLGVTVQVTIDP**A**stpapttptsttftPVTQEETSSSPTLTG 160

Isoform 7 78 DRVSIKDNQKNRTFTVTMEDLMKTDADTYWCGIEKTGNDLGVTVQVTIDP**A**--------------PVTQEETSSSPTLTG 143

Isoform 1 149 HHLDN-----------------------**R**HKLLKLSVLLPLIFTILLLLLVAASLLAWRMMKYQQK---**A**AGMSPEQVLQ 197

Isoform 2 159 HHLDNSssrdvprA-----------------------------------------------------------gtaAPGG 167

Isoform 3 150 HHLDNRSEGSQAANYRPAAHQAQAPEAQCPPAPHLHHIAAAFGGRLTLGLEDDEVPAESSRDVPRAgtaAPGGRPL---- 219

Isoform 4 164 HHLDN-----------------------RHKLLKLSVLLPLIFTILLLLLVAASLLAWRMMKYQQK---AAGMSPEQVLQ 211

Isoform 5 152 HHLDN-----------------------RHKLLKLSVLLPLIFTILLLLLVAASLLAWRMMKYQQK---AAGMSPEQVLQ 200

Isoform 6 167 HHLDN-----------------------RHKLLKLSVLLPLIFTILLLLLVAASLLAWRMMKYQQKgtaAPGGRPL---- 213

Isoform 7 149 HHLDN-----------------------RHKLLKLSVLLPLIFTILLLLLVAASLLAWRMMKYQQKgtaAPGGRPL---- 196

Isoform 1 204 PLEGDLCYADLTLQLAGTSPQKATTKLSSAQVDQVEVEYVTMASLPKEDISYASLTLGAEDQEPTYCNMGHLSSHLPGRG 277

Isoform 2 171 RPL--LCRPD---PAAGRN-------------------------LPAKGYHEAFLCPG---------------------- 195

Isoform 3 221 -----LCRPD---PAAGRN-------------------------LPAKGYHEAFLCPG---------------------- 244

Isoform 4 219 PLEGDLCYADLTLQLAGTSPQKATTKLSSAQVDQVEVEYVTMASLPKEDISYASLTLGAEDQEPTYCNMGHLSSHLPGRG 291

Isoform 5 207 PLEGDLCYADLTLQLAGTSPQKATTKLSSAQVDQVEVEYVTMASLPKEDISYASLTLGAEDQEPTYCNMGHLSSHLPGRG 280

Isoform 6 216 -----LCRPD---PAAGRN-------------------------LPAKGYHEAFLCPG---------------------- 239

Isoform 7 198 -----LCRPD---PAAGRN-------------------------LPAKGYHEAFLCPG---------------------- 221

Isoform 1 284 PEEPTEYSTISRP 290

Isoform 2 -------

Isoform 3 -------

Isoform 4 299 PEEPTEYSTISRP 305

Isoform 5 287 PEEPTEYSTISRP 293

Isoform 6 -------

Isoform 7 -------

Supplementary Figure 2
