# Supplementary material for: CD300f epitopes are specific targets for acute myeloid leukemia with monocytic differentiation
Source: Mol Oncol. 2019 Aug 20;13(10):2107–20. doi: 10.1002/1878-0261.12549 (PMC6763785; doi:10.1002/1878-0261.12549)
Supplement: Supplementary file 6 — Fig. S5. Cross‐blocking studies with CD300f antibodies. CD300f transfected CHO cells were incubated with a saturating amount of each primary antibody, (x axis). Cells were then stained with the test antibodies (A) UP‐D1 (B) 234903 (C) CLM‐1 (D) LMIR3. The binding of the test antibody in the presence of the primary antibody was calculated as by MFI compared to an isotype control, with 0 binding indicating complete overlap of epitopes, and 1 binding indicating no overlap of epitopes (n = 3). Error bars represent SEM. [file MOL2-13-2107-s006.pdf]

SUPPLEMENTARY MATERIAL

Supplementary Table 1. Clinical and Pathological Characteristics of AML Patient Samples Tested in the Current Study.

Sample ID	Age	Sex	Sample	WHO Diagnosis	Fab*	WCC	BM blasts %	Karyotype	NPM1	FLT3-ITD
1	77	F	PB	AML with MDS changes	M2	128	55	Normal	NT	NT
2	76	F	BM	AML NOS	M4	14.1	70	Normal	NT	NT
3	74	M	PB	AML NOS	M2	15.5	43	+8	NT	NT
4	70	M	BM	Therapy related myeloid neoplasm	M4	16.4	82	Normal	Detected	Detected
5	79	M	PB	Therapy related myeloid neoplasm	M4	150	67	46, XY, add(12)(q24.3)	NT	NT
6	89	M	PB	AML with MDS changes	M2	30.1	16	Normal	NT	NT
7	75	M	PB	RAEB-2	N/A	3.5	15	+8	NT	NT
8	59	M	PB	AML NOS	M5	146	95	Normal	NT	NT
9	51	M	PB	AML NOS	M0	2.5	73	No metaphases	Negative	Negative
10	59	M	PB	AML with MDS changes	M4	54.3	56	Normal	Negative	Detected
11	81	M	PB	CMML-2	N/A	5.7	10	Normal	NT	NT
12	86	F	PB	AML NOS	M1	23.2	NT	unknown	NT	NT
13	95	F	PB	AML with MDS changes	M5	62.7		unknown	NT	NT
14	82	M	PB	AML with MDS changes	M5	3.8	53	+19	NT	NT
15	61	F	BM	AML NOS	M6	0.7	26%	complex	NT	NT
16	16	M	BM	AML + inversion 16	M4Eo	111.3	45	Inversion 16	NT	NT
17	41	F	BM	AML NOS	M5	5.2	78	complex	NT	Negative
18	75	M	PB	AML with MDS changes	M2	6.5	60	Normal	NT	NT
19	73	F	PB	AML NOS	M0	8	85	Normal	Detected	Negative
20	72	F	PB	AML with MDS changes	M5	30.1	80	Complex;del(12)(p12),del(13)(q21), add(17)(p11.2)[1]46,XX[1]	NT	NT
21	59	M	BM	AML NOS	M0	14.1	60	Normal	Negative	Negative
22	23	M	BM	AML + inversion 16	M4Eo	160	60	46,XY,inv(16)(p13;q22)	Negative	Negative
23	87	M	PB	Therapy related myeloid neoplasm	M2	2.2	62	47,XY,t(8;16)(p11;p13), +8	NT	NT
24	24	M	PB	AML NOS	M5	64	90	t(11;17), +8	Negative	Negative
25	44	F	PB	AML + inversion 16	M4Eo	34.9	50	Inversion 16	Negative	Negative
26	79	M	BM	AML NOS	M2	54.6	60	+6	Negative	Negative
27	55	M	BM+PB	Blastic pDC Neoplasm	N/A	4.3	85	Normal	Negative	Negative
28	61	M	BM	AML NOS	M2	7	25	No metaphases	NT	NT
29	44	UD	BM	AML NOS	M1	UD	UD	t(9:11)	NT	NT
30	51	UD	BM	AML + inversion 16	M4Eo	UD	UD	inv 16	NT	NT
31	86	UD	BM	AML NOS	M4	UD	UD	+8	NT	NT
32	19	UD	BM	AML NOS	M2	UD	UD	monosomy 7	NT	NT
33	61	UD	BM	AML NOS	M4	UD	UD	Normal	NT	NT
34	34	M	PB	AML with Inv 16	M4eo	UD	UD	inv 16	NT	Negative

*M4/M5 are classed as monocytic AML, and all other subtypes as Non-monocytic AML.
